# Case report: Retrograde endovascular recanalization of vertebral artery occlusion with non-tapered stump via the deep cervical collateral

**DOI:** 10.3389/fneur.2023.1246151

**Published:** 2023-09-20

**Authors:** Han Qiu, Zhiming Kang, Dong Sun, Bin Mei, Junjian Zhang

**Affiliations:** ^1^Department of Neurology, Zhongnan Hospital of Wuhan University, Wuhan, Hubei, China; ^2^Hubei Province Clinical Research Center for Dementia and Cognitive Impairment, Wuhan, Hubei, China

**Keywords:** vertebral artery occlusion, non-tapered stump, ischemic stroke, endovascular recanalization, retrograde, deep cervical collateral

## Abstract

**Introduction:**

Vertebral artery (VA) occlusive disease is the major cause of posterior circulation ischemic stroke. Endovascular recanalization has been reported as a feasible treatment for patients with symptomatic VA occlusion refractory to optimal medical therapy. However, VA occlusion with non-tapered stump exhibits a low technique success rate when treated by antegrade endovascular therapy because of increased difficulty in passing the guidewire into the occluded segment. Herein, we presented a novel endovascular approach to recanalize chronically occluded VA with a non-tapered stump using a retrograde method *via* the deep cervical collateral, which has not been reported before.

**Case presentation:**

The present case was a patient with VA ostial occlusion with non-tapered stump and distal severe stenosis of the left VA who had recurrent posterior circulation transit ischemic attacks under optimal medical therapy. CT angiography demonstrated proximal non-tapered occlusion and distal severe stenosis of the left VA, and that the right VA did not converge with the left VA into basilar artery. Endovascular treatment was recommended and performed on this patient. However, antegrade endovascular recanalization of the left VA origin occlusion failed because the micro guidewire was unable to traverse the occluded segment. Fortunately, robust collateral from the deep cervical artery to the V3 segment of the left VA developed, in which we advanced the micro guidewire to the V3 segment of the left VA and reversely passed the micro guidewire through the occluded segment. Then, the occlusion and stenosis of the left VA were successfully resolved with angioplasty and stenting. After the procedure, the patient reported no neurological symptoms under medical therapy during 3-month follow-up.

**Conclusion:**

Antegrade endovascular recanalization of VA occlusion with a non-tapered stump is a challenge. The retrograde endovascular method *via* the cervical collateral may be an alternative for this type of VA occlusion, which requires further exploration.

## Introduction

Vertebral artery (VA) occlusive disease is a major cause of posterior circulation ischemic events, which accounts for approximately 20–32% of the transient ischemic attacks (TIAs) or ischemic strokes in the posterior circulation (1, 2). Atherosclerosis is the common etiology for this disease with the VA ostium as the most common site prone to be involved (3, 4). Current management of VA occlusive disease includes anti-thrombotic therapy, risk factor modification, open surgery, and endovascular treatment (5). Although the optimal strategy for preventing stroke occurrence in symptomatic patients with VA occlusion is controversial and empirical, the perspective that open surgery and endovascular treatment are important complementary treatments for symptomatic VA stenosis or occlusion refractory to optimal medical therapy seems to be well recognized (5–7). Open surgery including bypass surgery, vertebral endarterectomy, and hybrid surgery has been considered as a therapeutic option for VA occlusion (8–10), which, however, is not commonly performed due to the complexity and serious complications of these procedures (6). Recently, endovascular revascularization has been reported as a feasible treatment for VA occlusion with a high success rate of 86% and a low rate of periprocedural complications of 12% (5, 6). However, VA occlusion with a non-tapered stump exhibits a relatively low technique success rate of 71.4% when treated by antegrade endovascular revascularization because of increased difficulty in finding the access for the micro guidewire to pass through the occluded segment (6). In this report, we presented a novel approach to endovascular recanalize chronically occluded VA with a non-tapered stump using a retrograde method via the deep cervical collateral, which, to the best of our knowledge, has not been reported before.

## Case presentation

**Figure 1:**
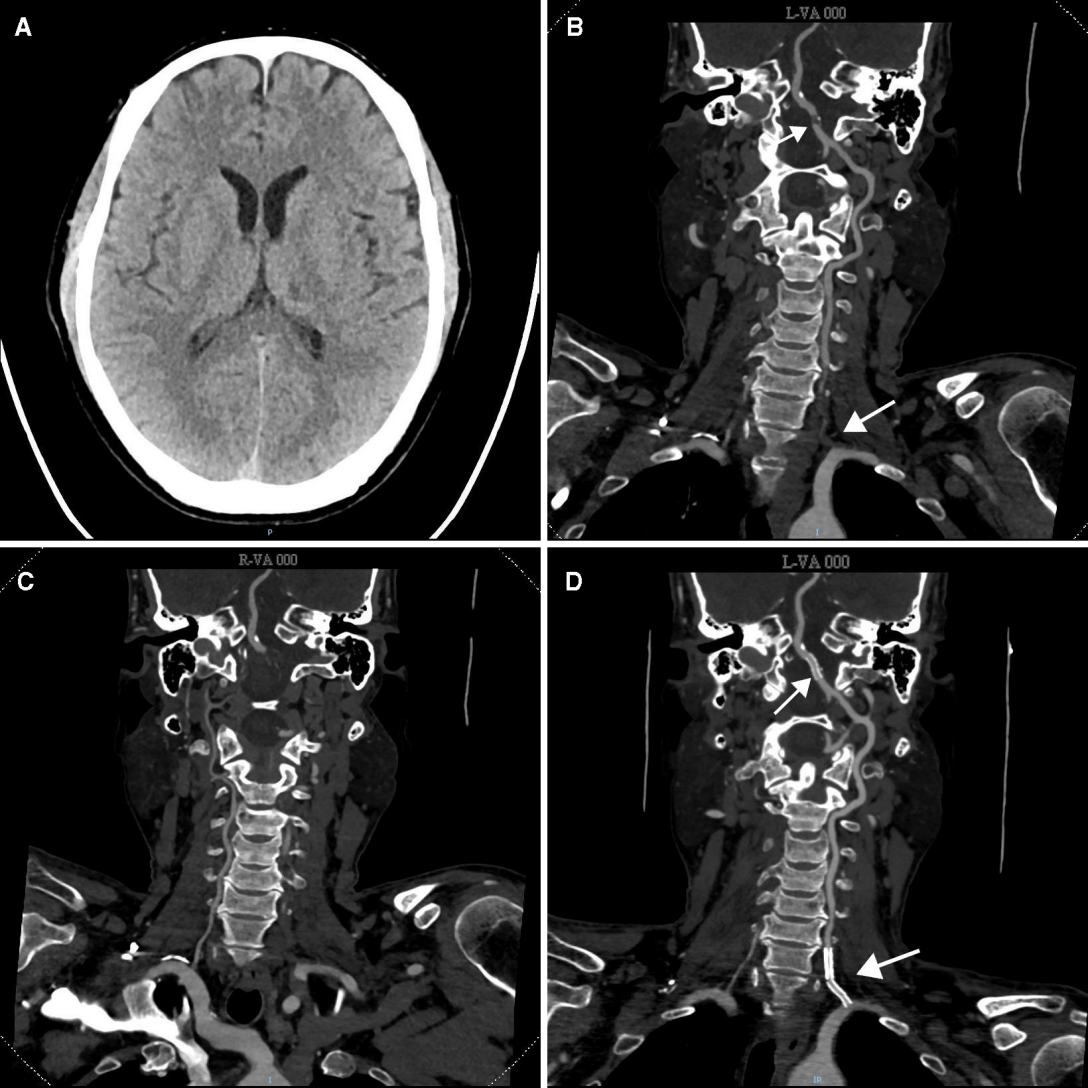
Pre- and post-treatment CT evaluation of the patient. **(A)** Pre-treatment non-contrast CT scan demonstrated a hypodensity lesion in the left thalamus. **(B)** Cervical and cerebral CT angiography (CTA) showed proximal occlusion with a non-tapered stump (white arrow) and distal severe stenosis of the left vertebral artery. **(C)** CTA showed that the right vertebral artery did not join in the basilar artery. **(D)** Post-treatment CTA indicated that the left vertebral artery was recanalized with stent placed inside (white arrow).

A 57-year-old man with a previous history of hypertension and stroke was referred to our hospital with transient episodes of dizziness, diplopia, and left-side numbness for 2 weeks. Each episode lasted for 5 to 30 min without unconsciousness and then completely relieved. After the onset of these symptoms, the patient was first admitted to a local hospital where he received optimal medical therapy including dual antiplatelet agents (aspirin 100 mg/day and clopidogrel 75 mg/day) and risk factor modifications. However, the patient still experienced another two episodes of TIAs under optimal medical treatment. His radiological examination in the local hospital suggested multifocal intracranial and extracranial atherosclerosis (data not shown). The patient was, therefore, transferred to our hospital for further management. After admission, the patient underwent a series of diagnostic evaluations. Neurological examination showed no permanent neurological disability with a National Institute of Health Stroke Scale score of 0. Laboratory tests including blood routine examination, hepatorenal function, lipid profile, homocysteine level, glycosylated hemoglobin level, and coagulation function were all normal. Multimodal computed tomography (CT) was also performed, and non-contrast CT demonstrated a hypodensity lesion in the left thalamus (Figure 1A). CT angiography (CTA) showed proximal non-tapered occlusion and distal severe stenosis of the left VA (Figure 1B), and that the right VA did not converge with the left VA into the basilar artery (Figure 1C). Based on these findings, the diagnosis of TIA due to VA occlusion was made. Since the patient had recurrent neurological symptoms despite optimal medical management, endovascular recanalization of the left VA was recommended.

**Figure 2:**
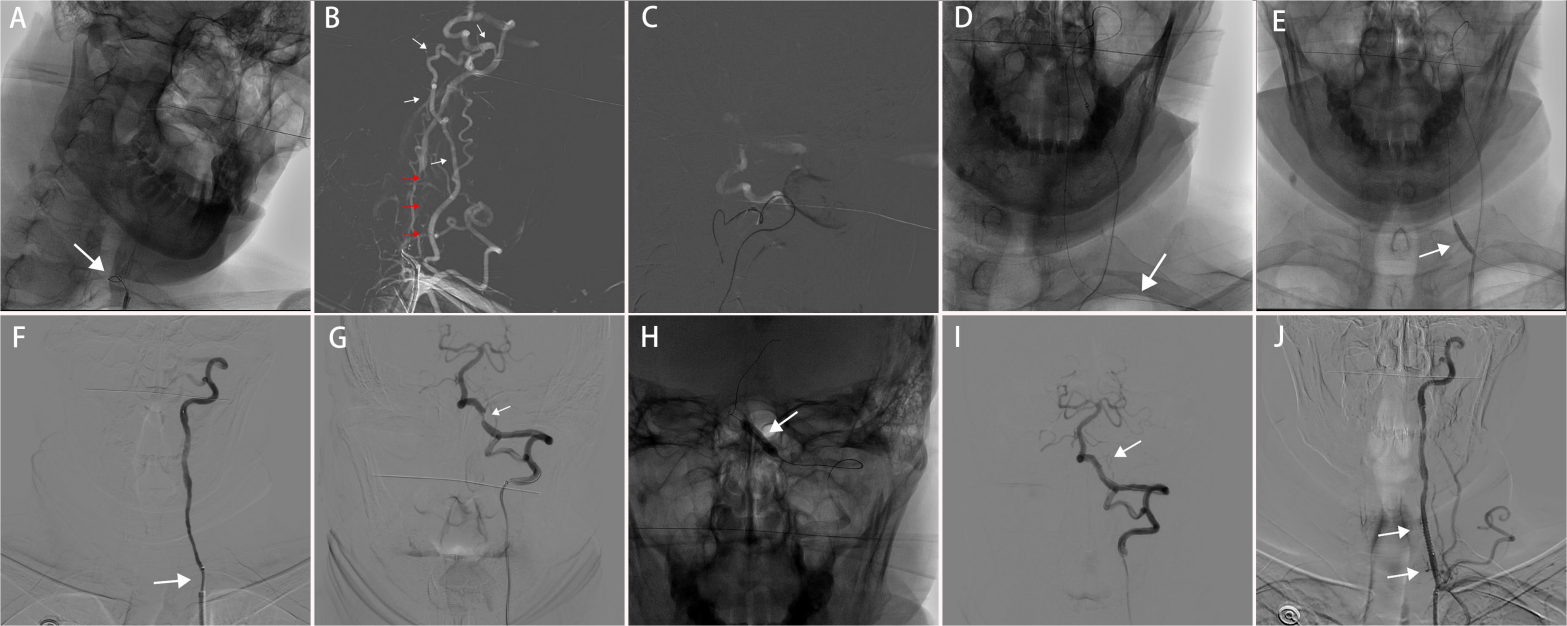
Endovascular procedure of the patient. **(A)** The micro guidewire was unable to across the occluded segment of the left vertebral artery. **(B)** Angiogram showed abundant collateral from the left deep cervical artery to the V3 segment of the left vertebral artery (white arrow), a tapered stump in the distal part of the occluded segment of the left vertebral artery (red arrow). **(C, D)** The micro guidewire reversely traversed the occluded segment of the left vertebral artery via the left deep cervical artery and was placed in the left subclavian artery [white arrow in **(D)**]. **(E)** Balloon dilation of the left vertebral artery occlusion (white arrow). **(F)** Occluded left vertebral artery was successfully reopened but remained with severe stenosis (white arrow). **(G)** Angiogram showed severe stenosis of the V4 segment of the left vertebral artery (white arrow). **(H)** A balloon-expandable stent was implemented in the stenotic segment of the left vertebral artery (white arrow). **(I)** After stenting, the stenosis of the V4 segment has been relieved (white arrow). **(J)** Stent placement of the V1 segment of the left vertebral artery to prevent restenosis (white arrow).

The procedure was performed under local anesthesia. After the femoral artery puncture, an 8F artery sheath was inserted, and the patient was intra-arterially heparinized to achieve an activated clotting time of more than 250 s. An 8F guiding catheter (Cordis, Florida, USA) and a 5F diagnostic catheter (Cordis, Florida, USA) were delivered to the left subclavian artery proximal to the VA ostium under the guidance of a 0.035-in loach guidewire (Terumo, Tokyo, Japan). After retracting the loach guidewire, an initial digital subtraction angiography (DSA) was performed *via* the diagnostic catheter, which demonstrated non-tapered occlusion of the V1 segment and severe stenosis (approximately 80%) of the V4 segment of the left VA. In the beginning, multiple attempts were performed with the coaxial assembly of a PT 0.014-in micro guidewire (Boston Scientific, Boston, USA) and an Excelsior SL-10 microcatheter (Stryker, Michigan, USA) to facilitate navigation across the occluded segment but failed (Figure 2A). At the moment, robust deep cervical collateral to the distal V3 segment of the left VA and a tapered stump of the distal part of the occluded segment were noted (Figure 2B). Therefore, the exchange of a Synchro 0.014-in micro guidewire (Stryker, Michigan, USA) was performed to reach the distal V3 segment through the left deep cervical artery, which then reversely traversed the occluded segment to the left subclavian artery successfully (Figures 2C, D). A Neuro RX 2.75 × 15 mm balloon (Sinomed, Tianjin, China) was subsequently advanced to the VA ostium along the micro guidewire to dilate the occluded segment (Figure 2E). After balloon dilation, the occluded segment was successfully recanalized but remained in severe stenosis (Figure 2F). We retracted the balloon and advanced a 5F intermediate catheter (Tonbridge, Zhuhai, China) with a Transend 0.014-in micro guidewire inside (Stryker, Michigan, USA), which passed the recanalized segment and was placed in the left VA and posterior cerebral artery. DSA revealed severe stenosis of the V4 segment and antegrade filling of the basilar artery and both posterior cerebral arteries (Figure 2G). Under the guidance of an angiogram, a NOVA 4.0 × 15 mm balloon-expandable stent (Sinomed, Tianjin, China) was implemented in the stenotic segment (Figures 2H, I). To prevent restenosis of the V1-V2 segment, a RX 4.0 × 18 mm balloon-expandable stent (Abbott, California, USA) and a Bridge 4.0 × 18 mm rapamycin drug-eluting stent (Microport, Shanghai, China) were consecutively implanted in the recanalized segment (Figure 2J). Finally, the 8F guiding catheter, intermediate catheter, and micro guidewire were carefully retracted.

**Figure 3:**
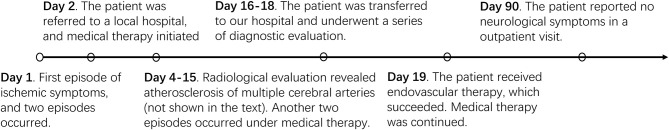
The timeline of the present case.

After the procedure, the patient underwent a repeated CTA examination, which indicated successful recanalization of the left VA (Figure 1D). Dual antiplatelet therapy and risk factor control strategies were continued in this patient. During the follow-up of 3 months, the patient reported no neurological symptoms. Figure 3 summarizes the timeline of the present case.

## Discussion

VA occlusive disease is a common cause of TIAs or strokes in the posterior circulation (1, 2), and VA ostial occlusion accounts for approximately 9% of posterior circulation infarcts (11). The mechanisms of ischemic cerebrovascular events caused by VA occlusive disease include hemodynamic impairment and vertebral artery stump syndrome (12, 13). Based on the angiographic findings, we speculated that the mechanism of the recurrent neurological symptoms of ischemia in the present patient was hemodynamic impairment due to VA occlusive disease. Although the patient did not undergo perfusion examination, DSA demonstrated antegrade filling of the basilar artery, which suggested that the source of blood supply to the posterior fossa might mainly come from the abundant collateral from the deep cervical artery. However, severe stenosis of the V4 segment of the left VA limited blood flow to the posterior fossa, and the right VA did not converge with the left VA into the basilar artery, which resulted in hemodynamic impairment. Therefore, relieving the stenosis of intracranial VA was the key to improve the hemodynamics and alleviate symptoms in this patient.

The two main strategies to regain the blood supply to the posterior fossa from an occluded VA are open surgery and endovascular treatment, which have been considered as complementary alternatives for patients with recurrent ischemic events under optimal medical therapy (6). Open surgery is limited to be performed in clinical practice because of technical complexity and high rates of morbidity and mortality (6, 14). Recently, several reports have evaluated the efficiency and safety of endovascular recanalization for symptomatic VA occlusion (5, 6, 14, 15). The technique success rate of endovascular recanalization of VA occlusion ranges from 86.0 to 91.3%, and the rate of periprocedural complications was 4.3–12.0% (5, 6), suggesting the feasibility of this treatment. Since the patient had recurrent neurological symptoms under optimal medical treatment, endovascular treatment was recommended. However, we encountered challenges in navigating the occluded segment of the non-tapered VA occlusion with a micro guidewire, which was the first step to recanalize the artery. This was not surprising as recent studies demonstrated that the stump morphology is a strong predictor of successful recanalization for extracranial VA occlusion, where the technique success rate of an occlusion with a tapered stump is significantly higher than that of a non-tapered stump occlusion (6). A non-tapered stump might hamper the micro guidewire to find the access to cross the occluded segment. Hence, it has long been a challenge to antegrade endovascular recanalization of a non-tapered occlusion.

Fortunately, the development of robust collateral flow from the deep cervical artery arising from the subclavian artery provided a new path for the micro guidewire to reversely cross the occluded segment in this patient. During the natural course of VA occlusion, collateral circulation may develop from the cervical arteries to the V2–V3 segment of VA (16), which could serve as an alternative routine to endovascular recanalization of the non-tapered VA occlusion when antegrade navigation failed. In a previous report, Ji et al. performed endovascular intervention for a patient with vertebral artery stump syndrome using retrograde recanalization *via* the ascending cervical artery when attempts of antegrade recanalization failed (17). Antegrade endovascular recanalization of a non-tapered occlusion is challenging due to morphologic and hemodynamic factors described above, where blood flow from the brachiocephalic trunk to the distal right subclavian artery may divert from the original direction of the micro guidewire. While the distal part of the occluded segment may have a tapered stump, the blood flow is usually static. The retrograde endovascular method takes advantage of morphologic and hemodynamic factors to recanalize a non-tapered occlusion. Thus, our report, together with the report Ji et al., suggested that the cervical collateral to the VA might be a new solution for endovascular recanalization of non-tapered VA origin occlusion. This, however, depends on the extent of the collateral developed.

Instead of recanalizing the occluded segment, antegrade recanalization of the stenosis of the V4 segment via the deep cervical artery was also theoretically possible, which, however, was not considered due to the acute tortuosity at the junction between the two vessels and the difficulty in delivering balloon and stent to the diseased site via the deep cervical artery. In addition, if the endovascular treatment had been a failure in this patient, open surgery would be considered.

## Conclusion

Antegrade endovascular recanalization of VA occlusion with a non-tapered stump has long been a challenge due to morphologic and hemodynamic factors. The retrograde endovascular method *via* the cervical collateral may be an alternative for this type of VA occlusion when antegrade recanalization fails, which requires further exploration.

## Patient perspective

The perspective of the patient was obtained in an outpatient visit during a follow-up of 3 months. The patient appreciated the doctors for solving his symptoms by endovascular recanalization of the occluded VA. He was amazed at the unique technique that the neurointerventionist performed. In addition, the patient agreed to publish his de-identified medical records.

## Data availability statement

The raw data supporting the conclusions of this article will be made available by the authors, without undue reservation.

## Ethics statement

The studies involving human participants were reviewed and approved by the Medical Ethics Committee of Zhongnan Hospital of Wuhan University (2023070K). The patients/participants provided their written informed consent to participate in this study. Written informed consent was obtained from the patient for the publication of any potentially identifiable images or data included in this article.

## Author contributions

HQ: conceptualization and design, literature review, and manuscript preparation. ZK: data collection, literature review, and manuscript preparation. DS: patient management and manuscript review. BM: patient management, manuscript review, and funding acquisition. JZ: manuscript review and supervision. All authors contributed to the article and approved the submitted version.
